# A Game-Based Computing Resource Allocation Scheme of Edge Server in Vehicular Edge Computing Networks Considering Diverse Task Offloading Modes

**DOI:** 10.3390/s24010069

**Published:** 2023-12-22

**Authors:** Xiangyan Liu, Jianhong Zheng, Meng Zhang, Yang Li, Rui Wang, Yun He

**Affiliations:** 1 School of Communications and Information Engineering, Chongqing University of Posts and Telecommunications, Chongqing 400065, China; zhengjh@cqupt.edu.cn (J.Z.); d190101012@stu.cqupt.edu.cn (R.W.); heyun@cqupt.edu.cn (Y.H.); 2 State Key Laboratory of Block Chain and Data Security, Zhejiang University, Hangzhou 310058, China; zhangmengyang@zju.edu.cn; 3 Cyberspace Security Key Laboratory of Sichuan Province, Chengdu 610043, China; liyang.yalee@gmail.com; 4 Department of Electronic Communication Engineering, Yuxi Normal University, Yuxi 653100, China

**Keywords:** vehicular edge computing networks, computing resource allocation, offloading strategy, exact potential game

## Abstract

Introducing partial task offloading into vehicle edge computing networks (VECNs) can ease the burden placed on the Internet of Vehicles (IoV) by emerging vehicle applications and services. In this circumstance, the task offloading ratio and the resource allocation of edge servers (ES) need to be addressed urgently. Based on this, we propose a best response-based centralized multi-TaV computation resource allocation algorithm (BR-CMCRA) by jointly considering service vehicle (SeV) selection, offloading strategy making, and computing resource allocation in a multiple task vehicle (TaV) system, and the utility function is related to the processing delay of all tasks, which ensures the TaVs’s quality of services (QoS). In the scheme, SeV is first selected from three candidate SeVs (CSVs) near the corresponding TaV based on the channel gain. Then, an exact potential game (EPG) is conducted to allocate computation resources, where the computing resources can be allocated step by step to achieve the maximum benefit. After the resource allocation, the task offloading ratio can be acquired accordingly. Simulation results show that the proposed algorithm has better performance than other basic algorithms.

## 1. Introduction

Vehicles will be highly connected with the aid of ubiquitous wireless networks. At the same time, vehicular applications and services, such as virtual reality, augmented reality, mixed reality, location-sharing applications, and sensor data-sharing applications, etc., are usually not only data-hungry but also computation-intensive and delay-sensitive, which is undoubtedly a massive burden on VECNs [1]. Fortunately, vehicle-to-everything (V2X) communications enable the process of transmission and computation from vehicles to other vehicles via vehicle-to-vehicle (V2V) links or to the infrastructure via vehicle-to-infrastructure (V2I) links. They are promising techniques for future vehicular applications providing high-transmission capacity and fast-computation capability experiences [2]. Based on V2X communications, the goal of applications and services processing among different entities can be achieved, which is helpful to partially reduce the traffic and computing load of VECNs.

To enable large-scale and ubiquitous automotive network access, traditional V2X technologies are evolving to IoV for increasing demands on emerging advanced vehicular applications [3]. As specified in 3GPP Release 14, V2X has two transmission modes in the cellular network [4]. The first is the direct mode, which includes V2I communication and V2V communication by using the dedicated side-link channel in the intelligent transportation systems (ITS) 5.9 GHz spectrum. The second is the vehicle-to-network communication in the mobile licensed spectrum [5]. Vehicular edge computing (VEC) achieved through V2X can significantly control end-to-end latency, which is a dominating advantage for vehicular services. Generally, vehicular services have different QoS requirements. To satisfy these requirements, wireless resource and computation resource allocation are challenges.

The authors in [6] elaborate, compare, and analyze the works in each mode (i.e., V2V, V2I, and V2X) according to the uplink data offloading for delay-sensitive data. V2V data offloading refers to transmitting data from TaVs to SeVs, which can leverage the computation resources of both TaVs and SeVs. Similarly, V2I offloading schemes can help when network connectivity is scant or lacks end-to-end connectivity. With the increasing number of vehicles and their services, V2X-based data offloading schemes are an encouraging way to address the tremendous traffic and computing burden of IoV. In addition, according to [7], data can be simultaneously offloaded to different entities, which means that a TaV can perform both V2V and V2I communication simultaneously.

Much work is focused on V2X-based task offloading schemes. The computing resources of SeVs are used via V2V links in [8,9,10,11,12]. Partial offloading is adopted in [8,9], which means the authors in [8,9] adopt Loc + SeV mode. Namely, tasks can be executed in multiple vehicle at the same time. The work in [10,11,12] all adopt 0–1 offloading, which means a task can only be executed locally or in a SeV. Moreover, the tasks in studies [10,11] can be offloaded to multiple SeVs. The difference is that [11] considers the velocity of a TaV, whereas [10] does not. The authors in [13,14,15,16] leverage the computation resources of ESs via V2I links. Where [13] is in Loc + Edge mode and adopts partial offloading, refs. [14,15,16] adopt 0–1 offloading. In addition, considering vehicles’ speed, the system in [14] is modeled in a time-discrete manner. Studies [15,16] do not consider the mobility of vehicles; ref. [15] considers the probability of task offloading. To fully use the computing resources near a TaV, refs. [17,18,19] jointly consider V2V and V2I links. Studies [17,18] adopt 0–1 offloading and tasks can only be executed in one of the three entities, i.e., Loc, SeV, and ES, whereas [19] adopts partial offloading and considers two execution modes, i.e., Loc + SeV mode and Loc + Edge mode.

In this paper, to further leverage the resources near a TaV, we consider four execution modes, namely, Loc execution mode, Loc + SeV execution mode, Loc + Edge execution mode, and Loc + SeV + EdgeV execution mode, where SeV can be a moving or stopped vehicle. When the ratio of tasks executed locally is 100%, the latter three modes become the Loc execution mode. Therefore, the Loc execution mode is listed separately to distinguish. In order to realize the minimization of processing delay and the maximization of accommodated TaVs, we propose a novel partial offloading and adaptive computation resource allocation scheme for VEC-assisted V2X networks by jointly optimizing SeV-selection factors, mode-selection factors, offloading ratios, and computation resource allocation coefficients. The main contributions of this paper are summarized as follows.

1. To maximize the overall benefits of the system, both SeVs and ES are used to provide computation resources via V2V links and V2I links, respectively, in the proposed slow-moving vehicle environment, where four offloading modes are studied, namely, Loc mode, Loc + SeV mode, Loc + SeV + EdgeV mode, and Loc + SeV + EdgeV mode. Based on these offloading modes, the optimal task offloading strategies and resource allocation strategies are realized by selecting SeVs, the offloading strategies development, and the ES computing resources allocation.
2. To reduce the impact of transmission time via V2V links on task processing time, the SeV is selected with the highest channel gain among three CSVs close to a TaV. When the SeV, execution mode, and the proportion of allocated ES computing resources are determined, if and only if the processing time of each execute terminal is the same can they reach the maximization system benefit. Based on this, the expression of the offloading ratio is derived.
3. To allocate computing resources of ES to the TaV that can bring the greatest gain to the system, a potential game based on pre-allocation is proposed, in which the maximum number of iterations is determined first. Then, the best response is used to achieve the best benefit step by step, and the allocated computation resources ratio and offloading strategies can be acquired accordingly. Additionally, the convergence of the proposed game is analyzed.
4. To verify the effectiveness of our scheme, we compare it with four based schemes, i.e., Loc + SeV Execution algorithm (LSVE) [8], Loc + ES Execution algorithm (LESE) [13], Loc + SeV and Loc + ES Execution algorithm (LSVLESE) [19], and Local Execution Algorithm (LEA). The results corroborate the superior performance of our proposed scheme.

The remainder of this paper is organized as follows. Section 2 reviews related work. In Section 3, we describe the model and define the relevant functions to formulate the task offloading and computing resources allocation problem. Section 4 introduces a centralized cooperative computation offloading game model, and the BR-CMCRA algorithm is used to find the Nash equilibrium (NE) point. Section 5 evaluates the performance and discusses the numerical results. Finally, Section 6 concludes this paper and discusses future work.

## 2. Related Work

The emergence of intelligent applications produces the demand for computing. Hence, how to reduce the computation pressure in VEC under massive computation demand is an urgent problem to solve. As mentioned above, SeVs and ES can share their idle computing resource to help TaVs deal with their tasks and provide low-latency computing services. Generally, applications are not processed only on restricted local resources while SeVs or ES are available. Much research has focused on the auxiliary computation of TaV at these two ends.

Due to the dynamic vehicular environment and the variation of available vehicular computing resources, it is a great challenge to design a practical task offloading mechanism to utilize vehicular computing resources via V2V links efficiently. The authors in [8] consider a three-node scenario where a source node wants to communicate with a destination node with the help of a relay node, where the relay node is for decoding and forwarding. The goal of the scenario is to optimize partitioning by a virtual queue model. Specifically, they present a stochastic modeling for V2V communication dynamics, an analytical model for characterizing the reliability of a V2V link, and an evaluation model to illustrate the computation reliability, which is defined as the probability that a vehicle successfully calculates a certain amount of data within a deadline. An offloading strategy for a vehicular fog computing-assisted platoon system is proposed in [9]. A task arrive at a TaV in the platoon according to the Poisson distribution. If the available resources in the platoon are sufficient, the TaV will request to offload the task to one SeV in the platoon. Otherwise, if the vehicular fog has sufficient computation resource, the TaV will transmit the task to the leader vehicle of the platoon, then the leader vehicle divides the task into the corresponding number of subtasks and transmits the subtasks to the corresponding SeVs in the vehicular fog one by one. The work in [10] considers a vehicle that has several computational tasks to process, but the limited onboard computational capability cannot satisfy the requirement of all tasks. It can offload some tasks to one neighboring vehicle with idle computing resources. In addition, the V2V communication link’s dynamic and vehicles’ computing resource allocation are considered. The work in [11] investigates the computation task allocation among vehicles with the help of a base station (BS) and proposes a distributed V2V computation offloading framework. In particular, the work formulates the task allocation problem as a sequential decision-making problem. Considering that vehicles with idle computing resources may not share their computing resources voluntarily, the work thus proposes a dynamic pricing scheme that motivates vehicles to contribute their computing resources according to the price they receive. The BS is used for information management and resource allocation. Similarly, the work in [12] leverages the ability of BSs to achieve inter-region task offloading and takes the role of BSs as the resource retailer in a proposed V2V trading paradigm. Based on the paradigm, the work proposes a distributed dynamic many-to-many task offloading framework to improve the edge resource utilization in VECNs while considering privately owned vehicles’ individual serving and offloading intentions.

ES deployed at a BS or a roadside unit (RSU) can also participate in computation to enhance the task processing capabilities of TaVs. On the one hand, the ES can participate in the computation process alone. The authors in work [13] propose an efficient partial computation offloading and adaptive task scheduling algorithm, where the system-wide profit problem is decoupled into three parts. To minimize the task transmission delay, they first develop an asymptotically optimal channel allocation discipline of all vehicles with a given offloading ratio. Then, to derive the optimal offloading ratio, a convex optimization problem is formulated that can maximize the system-wide profit. Finally, the payoff policy for offloading services is determined to achieve the constructed non-cooperative game’s equilibrium by jointly considering TaV’s position and speed, nearby vehicles, the available number of ESs, availability of the requested service, etc. The work in [14] considers a joint ES selection and computation offloading optimization problem in multiservice VECNs. The problem is considered a sequential decision-making problem, because a TaV, under the coverage of multiple ES, can select the proper one by sequentially testing them one after another. At the same time, it can make sequential decisions for finding a proper offloading ratio to the ES. However, every decision taken by TaVs can alter the surrounding environment’s state. The V2V communications are exploited to be aware of the surrounding environment and the potential offloading ESs, leading to better decisions in terms of network selection and offloading. ESs have limited computing resources, and multiple tasks may be offloaded to one ES, which leads to a set of contradictions in the supply and demand of computing resources. Taking into account the distance between TaV and RSU, application and communication models, and muti-TaV competition for ES resources, the work in [15] proposes a multi-TaV non-cooperative computation offloading game of a VEC scenario, in which each TaV adjusts its offloading probability to achieve the maximum utility. The authors in [16] classify MEC servers into three categories, i.e., hot MEC, neutral MEC, and nonhot MEC. They consider the sequential dependency between components that make up the task and strive to realize vehicular task offloading through cooperation among VEC servers based on the hot zone analysis. Specifically, their goal is to provide a collaborative way to minimize vehicular application latency.

On the other hand, ES can participate in the computation process together with SeVs. The work in [17] provides three available offloading modes for TaVs, i.e., local computing, edge offloading, and V2V offloading while considering the channel allocation, V2V pairing, and offloading mode selection. At the same time, one TaV can only choose one offloading mode, and when multiple compute-intensive TaVs turn to the same SeV for help, they will share the computation resource equally. Except for the offloading mode involved in [17], the work in [18] considers two extra modes, i.e., TaV offloads the task to an RSU for processing and uses V2V migration to transfer the computing results, and TaV offloads the task to an RSU for processing and uses I2I migration to transfer the computing results. To fully exploit computation resources, the authors in [19] assume that TaVs can selectively offload partial tasks to the RSU with a MEC sever or SeVs by jointly considering the delay requirements of tasks, communication distance, and the computation capability of RSUs. Then, three subproblems, i.e., an offloading-matching subproblem, a channel allocation subproblem, and a task offloading subproblem, are solved by a tabu search-based matching algorithm, a graph coloring algorithm, and a variable substitution approach, respectively. Thus, the appropriate SeVs to offload the computation tasks of TaVs, channel allocation for vehicles, and computation resource allocation can be achieved.

It is quite a challenge to satisfy the delay requirements of emergency computation-intensive vehicle applications. To minimize the total task processing delay, the work in [20] divides vehicles into four sets according to whether they have task offloading requirements or provide task processing services, and it considers task processing flexibility by deciding for each vehicle to process its tasks locally, to offload the tasks to RSU via V2I connections, or to other vehicles via V2V connections. The work in [21] jointly considers VEC server selection, offloading strategy, computation resource allocation, and load balancing among VEC servers for a multi-user multi-server VEC system. Due to the fact that the task processing delay is short, moving vehicles may have high satisfaction, and the utility function as a satisfaction function should monotonically decrease with the delay of TaV. Moreover, because of the limitation of computation resources, offloading can be less efficient and result in overload if all vehicles select the same VEC server to offload their task. The logarithmic utility is used here to satisfy the delay and load balancing requirements. Work in [22] considers a fixed number of time periods and minimizes the average offloading delay below the following three modes: (1) V2V offloading: TaVs directly offload tasks to their SeVs with surplus computing resources in a distributed manner; (2) V2I2V offloading: When there are no SeVs, tasks are first offloaded to an RSU and then assigned to other vehicles in a centralized manner; (3) V2I offloading: Tasks are offloaded to RSUs for direct processing. A comparison of the characteristics and the pros and cons in the recent research is presented in Table 1.

## 3. System Model and Problem Formulation

### 3.1. System Model

**Figure 1:**
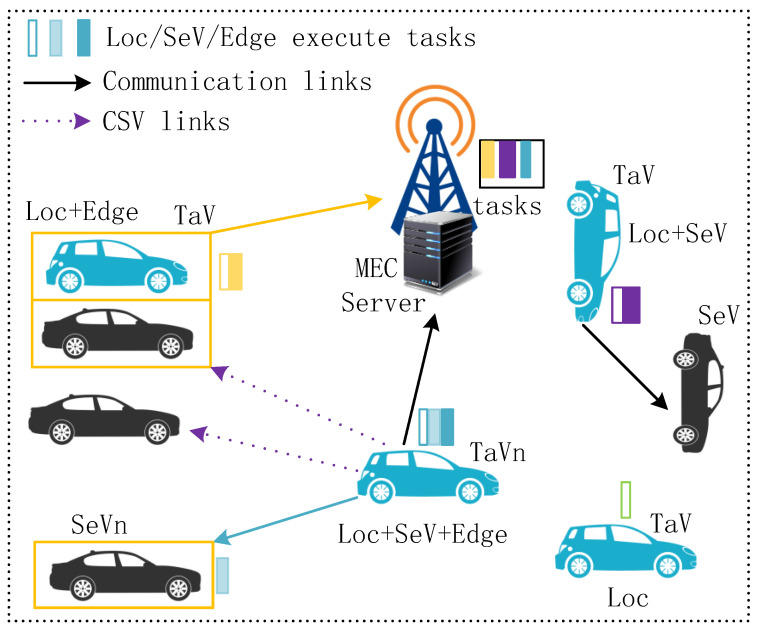
System model.

As presented in Figure 1, the system consists of an ES deployed at a BS/RSU and N TaVs with intensive tasks. Vehicles can be expressed as N={1,2,⋯,N}. In addition, task vehicle n is represented as TaVn and the profile of TaVn’s task as $I_{n}=\left\{D_{n},\mathrm{Ap}p_{n},\tau_{n}\right\}$, where the three items represent data size (in bits), task processing density (in CPU cycles/bit), and maximum tolerable latency (in seconds), respectively. The total CPU cycles required to complete the task is $C_{n}=D_{n}\mathrm{Ap}p_{n}$ [23]. Moreover, the channels of TaVs adopt orthogonal frequency division multiple access method. Each TaV has a corresponding SeV, namely, SeVn. Then, the SNR of TaVn to BS/RSU and to SeVn can be expressed as

(1) γnedg=PnedggnedgPnedggnedgδ2δ2,

(2) γnv2v=Pnv2vgnn′Pnv2vgnn′δ2δ2,

where $P_{n}^{edg}$ and $g_{n}^{edg}$ represent the transmitting power and channel gain of TaVn to BS/RSU. Similarly, $P_{n}^{v2v}$ and $g_{n}^{n^{\prime }}$ represent relevant parameters between TaVn and SeVn.

We adopt the urban channel model [24,25] for V2I and V2V links. The channel gain $g_{n}^{edg}$ from the TaV to the BS/RSU is modeled by

(3) gnedg=10(VehAntGain+BsAntGain−(PLV2I(dnedg)+SV2I)−BsNoiFig)(VehAntGain+BsAntGain−(PLV2I(dnedg)+SV2I)−BsNoiFig)1010,

where ${\mathrm{Veh}}_{\mathrm{Ant}}^{\mathrm{Gain}}$, ${\mathrm{Bs}}_{\mathrm{Ant}}^{\mathrm{Gain}}$, and ${\mathrm{Bs}}_{\mathrm{Noi}}^{\mathrm{Fig}}$ are vehicle antenna gain, BS/RSU antenna gain, and BS/RSU noise figure, respectively. The path-loss model $PL_{V2I}\left(d_{n}^{edg}\right)$ of the V2I links is [24]

(4) $$PL_{V2I}\left(d_{n}^{edg}\right)=128.1+37.6\mathrm{lo}g_{10}{(d}_{n}^{edg}/1000),\;\left(d_{n}^{edg}\;in\;m\right),$$

where $d_{n}^{edg}$ represents the distance between TaV and BS/RSU. The shadowing value $S_{V2I}$ denotes a Gaussian random value whose standard deviation is 8 dB.

Similarly, the channel gain between TaV and its SeV is modeled by

(5) gnn′=10(2∗VehAntGain−(PLV2V(dnn′)+SV2V)−VehNoiFig)(2∗VehAntGain−(PLV2V(dnn′)+SV2V)−VehNoiFig)1010,

where ${\mathrm{Veh}}_{\mathrm{Noi}}^{\mathrm{Fig}}$ represents vehicle noise. The path-loss models $PL_{V2V}\left(d_{n}^{n^{\prime }}\right)$ of the V2V links for line-of-sight conditions are given by [25]

(6) $$\begin{array}{c} PL_{V2V}\left(d_{n}^{n^{\prime }}\right)=\\ \left\{\begin{array}{c} 22.7*\log_{10}3+41+20*\log_{10}\left(freq/5\right),\;dist\leq 3\\ 22.7*\log_{10}\left(d_{n}^{n^{\prime }}\right)+41+20*\log_{10}\left(freq/5\right),\;d_{n}^{n^{\prime }}\leq 4*(H^{\mathrm{Veh}}{-1)}^{2}*freq/c\\ 40*\mathrm{lo}g_{10}\left(d_{n}^{n^{\prime }}\right)+9.45-17.3*\mathrm{lo}g_{10}((H^{\mathrm{Veh}}-1{)}^{2})+2.7*\log10(freq/5),\;\mathrm{else} \end{array}\right., \end{array}$$

where $d_{n}^{n^{\prime }}$, freq, and $H^{\mathrm{Veh}}$ represent the distance between TaV and SeVs, the carrier frequency, and vehicle antenna height, respectively. The standard deviation of $S_{V2V}$ is 3 dB. Thus, transmission rates from TaVn to BS/RSU and SeVn can be expressed as

(7) $$R_{n}^{edg}=B\log_{2}\left(1+\gamma_{n}^{edg}\right),$$

(8) $$R_{n}^{v2v}=B\log_{2}\left(1+\gamma_{n}^{v2v}\right).$$

### 3.2. Problem Formulation

The tasks of TaVn can be executed locally, offloaded to SeVn and to BS/RSU at the same time, and the proportion of each part can be expressed as $\alpha_{n}$, $\beta_{n}$, and $\theta_{n}$, which satisfy $\alpha_{n}+\beta_{n}+\theta_{n}=1,0\leq \left\{\alpha_{n},\beta_{n},\theta_{n}\right\}\leq 1$. They can also indicate the offload modes. In other words, when a task is not executed in one terminal, the corresponding offloading ratio is 0. The local execution time can be expressed as

(9) Tnloc=αnCnαnCnfnfn,

where $f_{n}$ is the local execution capacity. In particular, when all parts of tasks are executed locally, i.e., in Loc mode,$T_{n}=T_{n}^{loc}=C_{n}/f_{n}$, $\alpha_{n}=1$. In this case, $T_{n}$ is equal to the local execution time. Remote processing time includes transmission time and task execution time. In general, the size of the computational result is much smaller than the input data size of a task [26], and the send time is lower when one task needs to be offloaded. Then, the influence of downloading time and vehicles’ mobility is negligible, and the processing time of SeVn and ES can be expressed as

(10) tnn′=βnCnβnCnfn′fn′+βnDnβnDnRnv2vRnv2v,

(11) tnedg=θnCnθnCnρnFedgρnFedg+θnDnθnDnRnedgRnedg,

where $\rho_{n}F^{edg}$ represents the computing resources of ES allocated to TaVn, and $\rho_{n}$ is its indicator. When tasks are executed in TaVn and SeVn, the Loc + SeV mode is entered, and the processing time is the larger one of $T_{n}^{loc}$ and $T_{n}^{n^{\prime }}$, namely,

(12) Tn=Tnloc+sev=max{tnloc,tnn′}=max{αnCnαnCnfnfn,βnCnβnCnfn′fn′+βnDnβnDnRnv2vRnv2v}.

Due to the fact that tasks are not offloaded to ES, i.e., $\theta_{n}=0$, thus, $\alpha_{n}+\beta_{n}=1,\;0<\left\{\alpha_{n},\beta_{n}\right\}<1$. Similarly, when tasks are executed in local and ES, the Loc + Edge mode is entered, and the processing time is

(13) Tn=Tnloc+edg=max{tnloc,tnedg}=max{αnCnαnCnfnfn,θnCnθnCnρnFedgρnFedg+θnDnθnDnRnedgRnedg},

in the case, $\beta_{n}=0$, so, $\alpha_{n}+\theta_{n}=1,\;0<\left\{\alpha_{n},\theta_{n}\right\}<1$. The total processing time of Loc + SeV + EdgeV mode is the maximum one executed locally, offloaded to SeVn and to ES, which can be expressed as

(14) Tn=Tnloc+sev+edg=max{tnloc,tnn′,tnedg}=max{αnCnαnCnfnfn,βnCnβnCnfn′fn′+βnDnβnDnRnv2vRnv2v,θnCnθnCnρnFedgρnFedg+θnDnθnDnRnedgRnedg}

where $\alpha_{n}+\beta_{n}+\theta_{n}=1,0<\left\{\alpha_{n},\beta_{n},\theta_{n}\right\}<1$.

The total processing time of a TaV is related to its offloading ratio and the allocated computation resources of ES. We use $a_{n}=\left(\alpha_{n},\beta_{n},\theta_{n},\rho_{n}\right)$ to represent the action of a TaV, so the benefit based on the total time can be expressed as

(15) $$T_{n}\left(a_{n},a_{-n}\right)=\left\{\begin{array}{c} {-T}_{n}^{loc},\;\mathrm{if}\;\alpha_{n}=1,\;\beta_{n}=\theta_{n}=0\\ {-T}_{n}^{loc+sev},\;\mathrm{if}\;0<\{\alpha_{n},\beta_{n}\}<1,\;\theta_{n}=0,\;\alpha_{n}+\beta_{n}=1\\ {-T}_{n}^{loc+edg},\;\mathrm{if}\;0<\{\alpha_{n},\theta_{n}\}<1,\;\beta_{n}=0,\;\alpha_{n}+\theta_{n}=1\\ {-T}_{n}^{loc+sev+edg},\;\mathrm{if}\;0<\{\alpha_{n},\beta_{n},\theta_{n}\}<1,\;\alpha_{n}+\beta_{n}+\theta_{n}=1 \end{array}\right.,$$

where $a_{-n}$ denotes the joint action of others except for TaVn. Task processing time can reflect the system’s processing capacity to a certain extent, and lower processing time means higher processing capacity. Therefore, our goal is to maximize the total benefits of the system, which can be expressed as

(16) $$\begin{array}{c} P1\;\max\sum_{\left\{a_{n}\right\}}T_{n}\left(a_{n},a_{-n}\right)\\ s.t.\;\left(C1\right):\alpha_{n}+\beta_{n}+\theta_{n}=1,\;0\leq \left\{\alpha_{n},\beta_{n},\theta_{n}\right\}\leq 1,\;n\in N\\ \;\;(C2):0\leq \sum_{n\in N}\rho_{n}\leq 1,\;0<\rho_{n}\leq 1\\ \;\;(C3):{-T}_{n}\left(a_{n},a_{-n}\right)\leq \tau_{n},\;n\in N \end{array}$$

Constraint C1 ensures the integrity and separability of tasks. Constraint C2 ensures that the utilization of computing resources of ES does not exceed the maximum. Constraint C3 bounds the maximum delay of TaVn.

## 4. Problem Decomposition and Solution

### 4.1. Problem Decomposition

**Figure 2:**
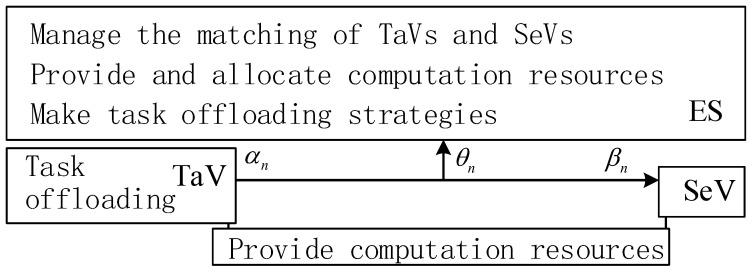
The relationship of TaV, SeV, and ES.

Figure 2 shows the relationship of the three terminals: TaV, SeVs, and ES. They all can provide computation resources. Moreover, the ES can manage the matching of TaVs and SeVs and make task-offloading strategies.

**Theorem** **1.** *The total task processing time is minimal only when the processing time of each execute terminal is the same.*

**Proof** **of Theorem 1.** Take the Loc + SeV + EdgeV mode as an example. Suppose there exist $\alpha_{n}^{\prime }$, $\beta_{n}^{\prime }$, and $\theta_{n}^{\prime }$ that make Tn=max{αn′Cnαn′Cnfnfn,βn′Cnβn′Cnfn′fn′+βn′Dnβn′DnRnv2vRnv2v,θn′Cnθn′CnρnFedgρnFedg+θn′Dnθn′DnRnedgRnedg} reach the minimal value, while satisfying

αn′Cnαn′Cnfnfn>βn′Cnβn′Cnfn′fn′+βn′Dnβn′DnRnv2vRnv2v>θn′Cnθn′CnρnFedgρnFedg+θn′Dnθn′DnRnedgRnedg,

it must be possible to adjust the ratio of $\alpha_{n}^{\prime }$, $\beta_{n}^{\prime }$, and $\theta_{n}^{\prime }$ to enable the task performed locally to decrease and the task performed remotely to increase so that the values of the three parts are gradually nearer and equal. Only if it satisfies

αn∗Cnαn∗Cnfnfn=βn∗Cnβn∗Cnfn′fn′+βn∗Dnβn∗DnRnv2vRnv2v=θn∗Cnθn∗CnρnFedgρnFedg+θn∗Dnθn∗DnRnedgRnedg, does $T_{n}$ reach the minimal value.    □

Therefore, when offloading modes and allocating ratio $\rho_{n}$ of TaVs are determined, the offload proportion can be directly obtained. To get the offloading ratio of Loc + SeV + EdgeV mode, we set

(17) αnCnαnCnfnfn=βnCnβnCnfn′fn′+βnDnβnDnRnv2vRnv2v=θnCnθnCnρnFedgρnFedg+θnDnθnDnRnedgRnedg,

and define

(18) Φ1=fnfnCnCnΦ2=Rnv2vfn′Rnv2vfn′(Rnv2vCn+Dnfn′)(Rnv2vCn+Dnfn′)Φ3=ρnFedgRnedgρnFedgRnedg(RnedgCn(RnedgCn+ρnFedgDn).

When we put (18) into (17), we have $\alpha_{n}\Phi_{1}=\beta_{n}\Phi_{2}=\theta_{n}\Phi_{3}$. Thus, the offloading ratio can be obtained by

(19) αn=Φ1Φ1(Φ1+Φ2+Φ3)(Φ1+Φ2+Φ3)βn=Φ2Φ2(Φ1+Φ2+Φ3)(Φ1+Φ2+Φ3)θn=Φ3Φ3(Φ1+Φ2+Φ3)(Φ1+Φ2+Φ3).

Similarly, the offloading ratio of Loc + SeV mode is denoted by

(20) αn=Φ1Φ1(Φ1+Φ2)(Φ1+Φ2)βn=Φ2Φ2(Φ1+Φ2)(Φ1+Φ2),

and the offloading ratio of Loc + Edge mode is denoted by

(21) αn=Φ1Φ1(Φ1+Φ3)(Φ1+Φ3)θn=Φ3Φ3(Φ1+Φ3)(Φ1+Φ3).

The point is to find the proper offloading mode, and when TaVn offloads its tasks to ES, $\rho_{n}$ is also needed. In addition, the purpose of finding a proper offloading mode and $\rho_{n}$ for a TaV is to maximize the benefit of the utility. In this process, each TaV should compete for computing resources and cooperate to maximize the overall benefit, which can be solved by a game.

### 4.2. Multiuser Computation Resource Allocation Game

We introduce game theory and construct a centralized framework to obtain an appropriate solution. Accordingly, we formulate the optimization problem as a multi-TaV task offloading game $G=\{N,A_{n},U_{n}\}$, where N is the TaVs’ set, $A_{n}=\alpha_{n}⊗\beta_{n}⊗\theta_{n}⊗\rho_{n}$ represents TaVn’s strategy space, and $U_{n}$ is the utility function of TaVn.

As in studies [17,27], the impact of a certain decision-making action on the whole system is measured by the marginal utility theory, and the utility function of a game participant is denoted by

(22) $$U_{n}\left(a_{n},a_{-n}\right)=T_{n}\left(a_{n},a_{-n}\right)+\sum_{i\neq n}\left(T_{i}\left(a_{i},a_{-i}\right)-T_{i}\left(a_{i},a_{-i\setminus n}\right)\right),$$

where $a_{-n}$ is the TaVs’ action profile, except TaVn, $T_{i}\left(a_{i},a_{-i}\right)$ is the benefit of TaVi when TaVn takes action, and $T_{i}\left(a_{i},a_{-i\setminus n}\right)$ is the benefit of TaVi when TaVn takes no action. Therefore, the first term and the second term of Equation (22) denote the benefit of TaVn and the influence on other TaVs brought by TaVn’s action, respectively.

The formulated game G reaches a NE while $a^{*}=\left(a_{1}^{*},a_{2}^{*},\ldots ,a_{N}^{*}\right)$ if and only if no TaV can increase its utility when changing its action unilaterally while other TaVs keep their decisions unchanged, namely,

(23) $$U_{n}\left(a_{n}^{*},a_{-n}^{*}\right)\geq U_{n}\left(a_{n},a_{-n}^{*}\right),\;\forall n\in N,\;\forall a_{n}\in A_{n},\;a_{n}\neq a_{n}^{*}.$$

In addition, if and only if there exists a potential function Θ such that [28]

(24) $$U_{n}\left({\tilde{a}}_{n},a_{-n}\right)-U_{n}\left(a_{n},a_{-n}\right)=\Theta \left({\tilde{a}}_{n},a_{-n}\right)-\Theta \left(a_{n},a_{-n}\right),\;\forall n\in N,\forall a_{n}\in A_{n},\forall {\tilde{a}}_{n}\in A_{n},$$

a game is an EPG, where $a_{n}$ and ${\tilde{a}}_{n}$ are available actions from TaVn’s strategy space. Equation (24) means that the fluctuation of the two functions is the same while any TaV changes its action.

**Theorem** **2.** *The proposed multi-TaV task offloading game is an EPG with at least one pure-strategy NE point, and the optimal combination of offloading strategy and computation resources allocation consists of a pure-strategy NE point of G.*

**Proof** **of Theorem 2.** Motivated by [17], to make the potential function have physical significance, the potential function is designed as

(25) $$\Theta \left(a_{n},a_{-n}\right)=\sum_{n\in N}T_{n}\left(a_{n},a_{-n}\right),$$

which is equivalent to the aggregate values of all TaVs. When TaVn changes its decision from $a_{n}$ to ${\tilde{a}}_{n}$, the change in individual utility function can be denoted by

(26) $$\begin{array}{c} U_{n}\left({\tilde{a}}_{n},a_{-n}\right)-U_{n}\left(a_{n},a_{-n}\right)\\ =T_{n}\left({\tilde{a}}_{n},a_{-n}\right)+\sum_{i\neq n}\left(T_{i}\left(a_{i},{\tilde{a}}_{-i}\right)-T_{i}\left(a_{i},{\tilde{a}}_{-i\setminus n}\right)\right)-(T_{n}\left(a_{n},a_{-n}\right)+\sum_{i\neq n}\left(T_{i}\left(a_{i},a_{-i}\right)-T_{i}\left(a_{i},a_{-i\setminus n}\right)\right))\\ =T_{n}\left({\tilde{a}}_{n},a_{-n}\right)-T_{n}\left(a_{n},a_{-n}\right)+\sum_{i\neq n}\left(T_{i}\left(a_{i},{\tilde{a}}_{-i}\right)-T_{i}\left(a_{i},a_{-i}\right)\right)+\sum_{i\neq n}\left(T_{i}\left(a_{i},a_{-i\setminus n}\right)-T_{i}\left(a_{i},{\tilde{a}}_{-i\setminus n}\right)\right) \end{array}.$$

Whether or not the action of TaVn changes, the influence on other TaVs maintaining their decisions is the same regardless of TaVn’s decision, i.e., $T_{i}\left(a_{i},a_{-i\setminus n}\right)=T_{i}\left(a_{i},{\tilde{a}}_{-i\setminus n}\right)$. Then, the above expression can be reorganized as

(27) $$\begin{array}{c} U_{n}\left({\tilde{a}}_{n},a_{-n}\right)-U_{n}\left(a_{n},a_{-n}\right)=T_{n}\left({\tilde{a}}_{n},a_{-n}\right)-T_{n}\left(a_{n},a_{-n}\right)+\sum_{i\neq n}\left(T_{i}\left(a_{i},{\tilde{a}}_{-i}\right)-T_{i}\left(a_{i},a_{-i}\right)\right)\\ =T_{n}\left({\tilde{a}}_{n},a_{-n}\right)+\sum_{i\neq n}T_{i}\left(a_{i},{\tilde{a}}_{-i}\right)-\left(T_{n}\left(a_{n},a_{-n}\right)+\sum_{i\neq n}T_{i}\left(a_{i},a_{-i}\right)\right)\\ =\Theta \left({\tilde{a}}_{n},a_{-n}\right)-\Theta \left(a_{n},a_{-n}\right) \end{array}.$$

Therefore, the constructed multi-TaV task offloading game is an EPG because the change in individual utility function is the same as in the potential function when any TaV unilaterally changes the decision. Based on the properties of EPG, the optimal solution constitutes a pure-strategy NE. Theorem 2 is proven.    □

### 4.3. Best Response-Based Centralized Multi-TaV Computation Resource Allocation Algorithm

In this section, we develop the BR-CMCRA algorithm to achieve the desirable NE point of the multi-TaV computation resource allocation game.

#### 4.3.1. Algorithm Design

The BR-CMCRA algorithm based on the best response is proposed here, and the algorithm follows one design principle of game theory, namely, the finite improvement property. Based on the property, the game player can improve its utility step by step. This principle is embodied in many learning algorithms, e.g., best response and better reply [29]. The best response can find the best solution in each iteration. Specifically, the chosen TaVn updates its decision if and only if it can improve more properties than the other TaVs. Otherwise, TaVn decides to keep its decision unchanged. The specific algorithm process is shown in Algorithm 1.
**Algorithm 1** BR-CMCRA algorithm**Initialization:**1. Each TaV chooses its SeV based on the channel gains from its nearest three candidate SeVs.2. The processing time of a TaV’s task is first initialized according to the time of Loc execution mode and Loc + SeV execution mode. Specifically, if the local execution time is smaller, it is initialized to the Loc mode. Otherwise, it is initialized to the Loc + SeV mode.3. The edge computing resources are divided into λN parts, and the ratio of each part is 11λNλN, where λ is a constant factor. The iteration index *k* of the proposed algorithm is set to 1.**Repeat Iterations:****Step 1:** One part computation resource FedgFedgλNλN is taken out and will be allocated in this iteration.**Step 2:** The ES maintains a table that stores the processing time of each TaV in different modes, i.e., Loc + SeV mode, Loc + Edge mode, Loc + SeV + EdgeV mode, and Loc execution mode.**Step 3:** Evaluate the completion status of each TaV and if $\Delta T_{n}<0$. The picked TaVn updates its allocated computation ratio $\rho_{n}$ based on the following rule:(28)$$a_{n}\left(k+1\right)=\left\{\begin{array}{c} a_{n}^{\prime },\;if\;\min\Delta T_{n}\\ a_{n}\left(k\right),\;\;else \end{array}\right.$$**Step 4:** If $\Delta T_{n}\geq 0$, evaluate the improved utility of each TaV according to the BS/RSU for an update opportunity. The picked TaVn updates its allocated computation ratio $\rho_{n}$ based on the following rule:(29)$$a_{n}\left(k+1\right)=\left\{\begin{array}{c} a_{n}^{\prime \prime },\;if\max\Delta U_{n}\\ a_{n}\left(k\right),\;else \end{array}\right.$$
where $a_{n}^{\prime }$ and $a_{n}^{\prime \prime }$ is the decision based on Equations (30) and (31) of TaVn; $a_{n}\left(k+1\right)$ is TaVn’s decision in the (*k* + 1)th iteration, while other TaVs keep their decision unchanged.**Step 5:** Update the offloading strategy based on Equations (19)–(21); k=k+1.The algorithm will terminate when the utility reaches the maximum number of iterations, i.e., λN.**End** 

It’s worth noting that the new action $a_{n}^{\prime }$, selected from the strategy space of player n, is updated if player n cannot complete processing its tasks, i.e.,

(30) $$a_{n}^{\prime }=\min\left(\left\{a_{n}^{\prime }|a_{n}^{\prime }\in A_{n},\Delta T_{n}\right\}\right),\;\forall n\in N,$$

where $A_{n}$ indicates TaVn’s strategy space, and $\Delta T_{n}=\tau_{n}-T_{n}\left(a_{n},a_{-n}\right)$ denotes the difference between the maximum delay and the processing time. Similarly, the new action $a_{n}^{\prime \prime }$ is updated if and only if $a_{n}^{\prime \prime }$ can bring the highest improved utility than the other players, i.e.,

(31) $$a_{n}^{\prime \prime }=\max\left(\left\{a_{n}^{\prime \prime }|a_{n}^{\prime \prime }\in A_{n},\Delta U_{n}\right\}\right),\;\forall n\in N,$$

where $\Delta U_{n}=U_{n}\left(a_{n}^{\prime },a_{-n}\right)-U_{n}\left(a_{n},a_{-n}\right)$ represents the improved utility.

We adopt a pre-allocation mechanism to achieve the improved benefit comparison between multi-TaV interactions. Specifically, a part of edge computing resource FedgFedgλNλN is pre-allocated to each TaV, and the processing time is calculated and stored in ES. Then, the improved benefits can be acquired based on the pre-allocated resources and the previous results.

#### 4.3.2. Analysis of the Convergence and Complexity

The aforementioned game model is an EPG, so all TaVs can gradually improve their utility step by step based on the finite improvement property [28]. In addition, the change of value in the constructed utility function and its corresponding potential function is just the same when any TaV changes its decision, i.e., $U_{n}\left({\tilde{a}}_{n},a_{-n}\right)-U_{n}\left(a_{n},a_{-n}\right)=\Theta \left({\tilde{a}}_{n},a_{-n}\right)-\Theta \left(a_{n},a_{-n}\right)$ in Equation (24). It means the optimization objective will improve with the rise of utility. Since the finite strategy space bounds the utility, if no TaV wants to change its current decision, the proposed algorithm will converge and achieve the maximization of the optimization problem locally or globally. In other words, the BR-DMCTO algorithm converges to the NE point of the optimization problem.

The complexity of the proposed algorithm is analyzed here. As shown in Algorithm 1, the number of iterations is λN. In each iteration, the complexity mainly comes from the ES computation resources selection of TaVs. In the first iteration, the TaV with the maximum increment utility is selected for updating. The complexity is log(N). In the subsequent iterations, only the TaV that allocated computation resources will update its increment utility before selection. The complexity is log(N1) or log(N2) based on steps 3 and 4, where N1 and N2 represent the TaVs that satisfy $\Delta T_{n}<0$ and $\Delta T_{n}\geq 0$, respectively. Let us consider the worst-case scenario where the complexity of each iteration is log(N). Hence, the complexity of the proposed algorithm is O(λN∗log(N)).

## 5. Performance Evaluation

Simulation results are given to verify the effectiveness of our proposed algorithm, which are averaged over 1000 independent experiments to ensure the scientific nature of the simulation. It is worth mentioning that if the velocity of vehicles is 30 (in m/s), then the average inter-vehicle distance is 2.5 × 30/3.6 according to TR 36.885, i.e., 20.83. In addition, the cell radius CR of the model is 500 (in m). The major simulation parameters are summarized in Table 2.

The performance of our proposed BR-CMCRA algorithm is compared with the following four algorithms about the total/completed processing delay, maximum processing time, average throughput of TaVs, number of devices executed in each mode, and lost rate of each execution mode.

1. LSVE [8]: TaVs leverage Loc + SeV mode to obtain the allocation strategies;
2. LESE [13]: TaVs leverage Loc + Edge mode to obtain the allocation strategies;
3. LSVLESE [19]:TaVs leverage Loc + SeV mode or Loc + Edge mode to obtain the allocation strategies;
4. LEA: All tasks are executed locally.

### 5.1. Convergence Behavior

We analyze the convergence of computation resource allocation from the perspective of the performance gap of the last two iterations. Algorithms requiring the assistance of ES include BR-CMCRA, LESE, and LSVLESE. As shown in Table 3, the gap of our proposed BR-CMCRA algorithm fluctuates around 0.01, which means the performance cannot improve a lot to some degree. In addition, the maximum gap of the LESE algorithm is 0.0312, which is acceptable. The LSVLESE algorithm’s gap is significant because its iteration times are five times the amount of TaVs, whereas the other algorithms are set to ten times. The iterations are fewer in the LSVLESE algorithm because when a task is chosen to be executed in Loc + Edge mode, the allocated computation resources must exceed a certain amount, or the TaV may prefer to choose Loc + SeV mode.

### 5.2. Performance Comparison

**Figure 3:**
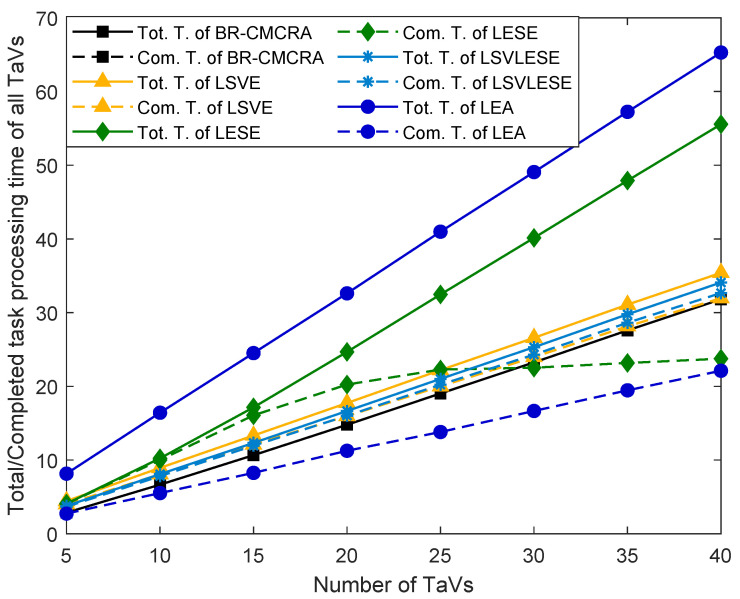
The total/completed task processing time versus the number of TaVs.

Figure 3 shows the total and completed task processing time of TaVs, and the total task completion time is the total task processing time minus the task processing time that cannot be completed. The proposed algorithm has the lowest value. The task processing time of all algorithms increases linearly except the completed time of the LESE algorithm. When the number of TaVs is fewer than 20, with the increase of tasks, the LESE algorithm’s task completion time gradually increases. Specifically, when the number of tasks is fewer than 15, there is little difference between the completed and total task processing time. In this case, ES can assist the TaVs in completing their tasks within a specified delay. When the number of tasks increases, the number of tasks that cannot be completed increases, and the computation resources of ES cannot provide enough services. Thus, the gap between the two times becomes larger and larger. When the number of tasks equals 40, the completed task processing time is slightly larger than that of LEA. Since TaV has limited resources, the two curves of the LEA algorithm are at the outermost. Specifically, LEA has the largest total task processing time and the smallest completed task processing time. Compared with LEA, the performance of LSVE has improved a lot, which means that SeVs help a lot. Furthermore, with the help of ES, the total processing time of the LSVLESE algorithm is smaller than LSVE, and the completed processing time of the two algorithms are the opposite. The two times of BR-CMCRA coincide, which means the tasks can be totally completed in our proposed algorithm.

**Figure 4:**
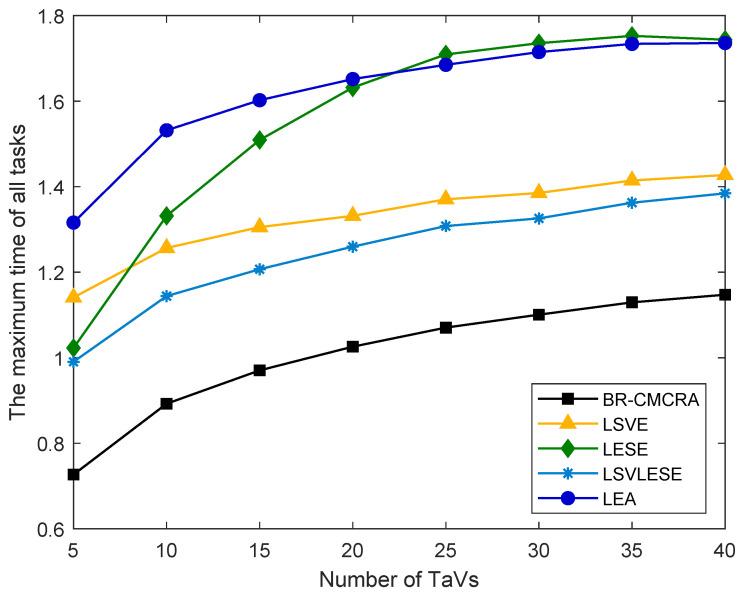
The maximum time of all tasks versus the number of TaVs.

The maximum task execution time is the maximum task execution time of those tasks that can be completed within the limited latency. As shown in Figure 4, the maximum time of our proposed BR-CMCRA algorithm is the minimal one. Except for the LESE algorithm, the curves of other algorithms are the same and do not cross each other. The LESE algorithm crosses with the LEA algorithm and the LSVE algorithm. As the tasks executed in ES increase, the computing resources of ES become insufficient, and the performance of the LESE algorithm will decline.

**Figure 5:**
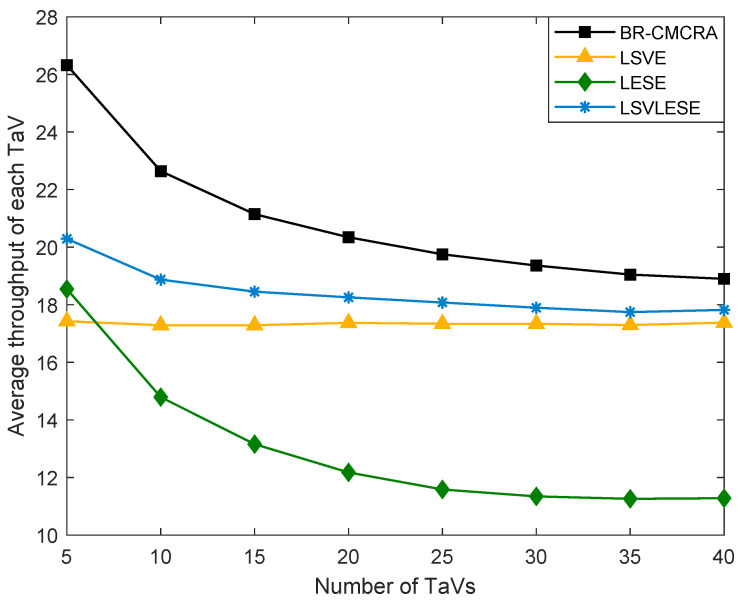
The average throughput of each TaV versus the number of TaVs.

As shown in Figure 5, the average throughput of each algorithm is given. As the LEA algorithm executes tasks locally, its throughput is constant zero. We disregard it in the figure. Because the number of TaVs and the number of SeVs of the LSVE algorithm increases 1:1, the throughput of each vehicle is relatively stable, which is from V2V links. Except for V2V links, LSVLESE also uses the V2I links; the throughput of the LSVLESE algorithm is larger than that of the LSVE algorithm. The BR-CMCRA and LESE algorithms’ trends are similar, which can reflect the influence of ES to a degree. In addition, the difference in values between the two algorithms can reflect the influence of SeVs to a degree.

**Figure 6:**
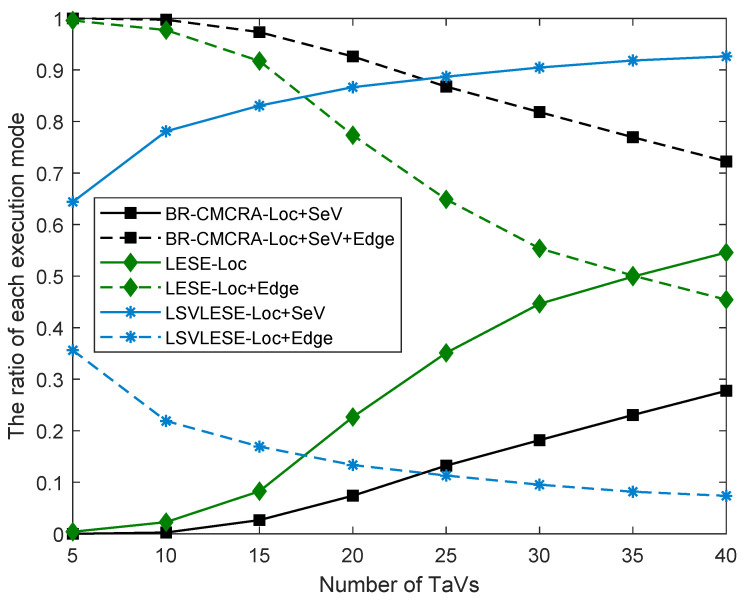
The ratio of each execution mode versus the number of TaVs.

As shown in Figure 6, the ratio of TaVs executing tasks in different modes are given, which can reflect the execution tendency of TaVs’ tasks under these algorithms. The LSVE algorithm and the LEA algorithm have only one execution mode. The execution ratio is 100%, so they are not drawn in Figure 6. The BR-CMCRA algorithm includes Loc + SeV execution mode and Loc + SeV + EdgeV execution mode, and tasks in the BR-CMCRA algorithm tend to be executed in Loc + SeV + EdgeV execution mode because tasks executed in more entities may improve performance. In addition, the two curves of the BR-CMCRA algorithm do not intersect, which means the Loc + SeV + EdgeV execution mode has obvious advantages. Similar to the BR-CMCRA algorithm, the two curves of the LSVLESE algorithm also do not intersect. The LSVLESE algorithm includes Loc + SeV execution mode and Loc + Edge execution mode, and tasks in the algorithm tend to be executed in Loc + SeV execution mode. The number of TaVs that ES can provide computation resources for is constant, and each TaV can be served by a corresponding SeV. Unlike the BR-CMCRA algorithm and the LSVLESE algorithm, the two curves of the LESE algorithm have a point of intersection. The LESE algorithm involves Loc execution mode and Loc + Edge execution mode. When the task number is less than 35, tasks tend to be executed in Loc + Edge mode. ES can provide services for TaVs at this time. However, when the task number is more than 35, tasks tend to be executed in Loc mode because ES’s computation resources are insufficient. When the task number is near 35, the ratio of the Loc execution mode is equal to that of the Loc + Edge execution mode.

**Figure 7:**
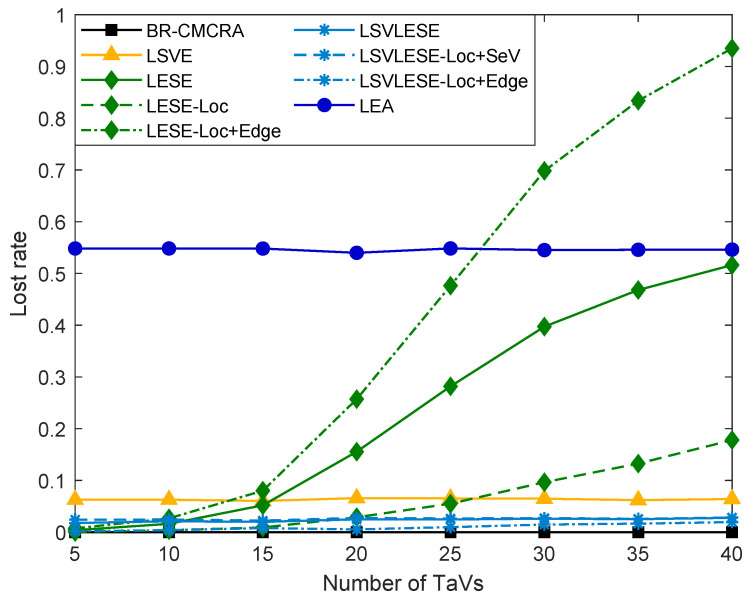
The lost rate versus the number of TaVs.

As shown in Figure 7, the lost rate of each execution mode in each algorithm is simulated. The lost rate of the BR-CMCRA algorithm is approximately equal to 0, which means the tasks of all TaVs can be executed within its delay. Compared with the BR-CMCRA algorithm, the LSVLESE algorithm changes Loc + SeV + EdgeV execution mode into Loc + Edge mode, causing the lost rate of the LSVLESE algorithm to be slightly higher than the BR-CMCRA algorithm. Without the help of ES, the lost rate of the LSVE algorithm is higher than the BR-CMCRA and LSVLESE algorithms and ranges from 1% to 2%. The LEA algorithm, which executes tasks locally, has the highest lost rate. In addition, the lost rate of the BR-CMCRA algorithm, LSVLESE algorithm, LSVE algorithm, and LEA algorithm are all relatively stable. On the contrary, the lost rate of the LESE algorithm increases rapidly, and that of the LESE algorithm in Loc + Edge execution modes even exceeds the LEA algorithm. Because ES fails to decrease the task processing time to less than limited latency, the lost rate of the LESE algorithm mainly comes from Loc + Edge. Similarly, the lost rate of the LSVLESE algorithm mainly comes from the Loc + SeV execution mode.

## 6. Conclusions

A best-centralized edge computing resource allocation scheme based on response is proposed, and a pre-allocation mechanism is adopted to select the computation resources for TaVs with the best benefit. However, the channel state information is relatively stable in our proposed scheme. In future work, we will consider the change of channel state information and offloading strategy caused by the mobility of vehicles. In addition, when TaVs have multiple task to be executed, the asynchronous offloading of multiple TaVs is worth considering.

## Figures and Tables

**Table 1:** Comparison with the latest related studies.

Ref.	Year	Loc	SeV	Edge	Velocity	Advantages	Shortcomings
[8]	2021	✓	✓	✗	✗	A virtual queue model is proposed to optimize partitioning	The velocity of vehicle is not given
[9]	2023	✓	✓	✗	✗	Both 0–1 offloading and partial offloading are considered	The mobility of vehicles is not considered
[10]	2022	✗	✓	✗	✗	Multiple SeVs can provide service for a TaV	Only one TaV is considered
[11]	2020	✗	✓	✗	✓	Relative velocity is considered and BS is used for information management	One TaV is considered
[12]	2022	✓	✓	✗	✓	Vehicles are motivated to form coalitions to operate the resources cooperatively	Channel model is not considered
[13]	2022	✓	✗	✓	✗	Non-orthogonal multiple access is considered	Does not consider the resources of nearby vehicles
[14]	2023	✓	✗	✓	✓	V2V links are used to be aware of the surrounding environment and the potential offloading of ESs	Each TaV can offload data to only one ES
[15]	2020	✓	✗	✓	✗	The offloading probability of TaVs are considered	Handover problem between different MEC platforms is not considered
[16]	2020	✓	✗	✓	✗	MEC servers are classified into three categories	Information management needs to be considered
[17]	2022	✓	✓	✓	✗	Both SeV and ES can provide services	Only one terminal can be chosen once
[18]	2022	✓	✓	✓	✓	V2V migration and I2I migration are used to transfer the computing results	Only one task of a TaV is considered
[19]	2023	✓	✓	✓	✓	Multiple offloading modes are considered	A computation task of a TaV is considered
[20]	2023	✓	✓	✓	✓	Vehicle velocity distribution is analyzed	The time-varying or stochastic V2V channel gain is not considered
[21]	2019	✓	✗	✓	✓	Integrating load balancing with offloading is proposed	The arriving vehicle has a constant speed
[22]	2019	✗	✓	✓	✗	Task is generated in time period and V2I2V offloading is considered	Local computing resources is not used
Ours		✓	✓	✓	✓	The offloading mode can be chosen adaptively	A TaV has only one task

**Table 2:** Simulator parameters.

Parameter	Value
Wireless bandwidth of the BS/RSU (B)	2 MHz
Data size of a task ($D_{n}$)	[10, 20] Mbits
The required CPU cycles per bit of a task ($\mathrm{Ap}p_{n}$)	[100, 200] CPU cycles/bit
The maximum tolerable delay of a task ($\tau_{n}$)	[1, 2] s
Transmit power of the vehicles (P)	23 dBm
CPU cycle frequency of the ES ($F^{edg}$)	10 GHz
CPU cycle frequency of a TaV ($f_{n}$) or a SeV ($f_{n^{\prime }}$)	[1, 2] GHz
Noise power ($\delta^{2}$)	−114 dBm
The cell radius (CR)	500 m
BS/RSU height	25
Vehicles antenna height ($H^{\mathrm{Veh}}$)	1.5
Carrier frequency (freq)	2 GHz
Antenna gain of BS/RSU and vehicles (${\mathrm{Bs}}_{\mathrm{Ant}}^{\mathrm{Gain}}$ and ${\mathrm{Veh}}_{\mathrm{Ant}}^{\mathrm{Gain}}$)	8 dBi and 3 dBi
The noise figure of BS/RSU and vehicles (${\mathrm{Bs}}_{\mathrm{Noi}}^{\mathrm{Fig}}$ and ${\mathrm{Veh}}_{\mathrm{Noi}}^{\mathrm{Fig}}$)	5 dB and 9 dB

**Table 3:** The convergence of computation resource allocation.

Number of Vehicles	5	10	15	20	25	30	35	40
BR-CMCRA	0.0129	0.0127	0.0112	0.0099	0.0089	0.0080	0.0073	0.0067
LESE [13]	0.0262	0.0312	0.0309	0.0276	0.0240	0.0201	0.0172	0.0155
LSVLESE [19]	0.2063	0.1875	0.1955	0.1038	0.2066	0.2081	0.2164	0.2131

## Data Availability

Data are contained within the article.

## Abbreviations

The following abbreviations are used in this manuscript:

VECNs	vehicle edge computing networks
IoV	Internet of Vehicles
ES	edge server
BR-CMCRA	a best response-based centralized multi-TaV computation resource allocation algorithm
SeV	service vehicle
TaV	task vehicle
QoS	quality of services
CSV	candidate SeVs
EPG	exact potential game
V2X	vehicle-to-everything
V2V	vehicle-to-vehicle
V2I	vehicle-to-infrastructure
ITS	intelligent transportation systems
VEC	vehicular edge computing
LSVE	Local + SeV execution algorithm
LESE	Local + Edge execution algorithm
LSVLESE	Local + SeV and Local + ES execution algorithm
LEA	Local execution algorithm
NE	Nash equilibrium
BS	base station
RSU	roadside unit
